# Features of the gut microbiota in ulcerative colitis patients with depression: A pilot study

**DOI:** 10.1097/MD.0000000000024845

**Published:** 2021-02-19

**Authors:** De-Liang Chen, Yan-Cheng Dai, Lie Zheng, You-Lan Chen, Ya-Li Zhang, Zhi-Peng Tang

**Affiliations:** a Institute of Digestive Diseases, LongHua Hospital, Shanghai University of Traditional Chinese Medicine, Shanghai; b Department of Tuina, Affiliated Hospital of Nanjing University of Chinese Medicine, Nanjing, Jiangsu Province; c Department of Gastroenterology, Shanghai Traditional Chinese Medicine-Integrated Hospital, Shanghai University of Traditional Chinese Medicine, Shanghai; d Department of Gastroenterology, Traditional Chinese Medicine Hospital of Shaanxi Province, Xi’an, Shaanxi Province; e Department of Gastroenterology, The First Affiliated Hospital of Guangzhou University of Chinese Medicine, Guangzhou, Guangdong Province, China.

**Keywords:** colitis, depression, *Firmicutes*, gastrointestinal microbiome, microbiota, *Proteobacteria*, ulcerative

## Abstract

Despite the establishment of the links between ulcerative colitis (UC) and depression, between UC and gut microbiota, few correlations between depression and gut microbiota have yet been demonstrated especially in ulcerative colitis patients. The objective of our study was therefore to determine whether the comorbidity of depressive disorder in ulcerative colitis patients correlate with alterations in the gut microbiota and to identify the specific microbiota signatures associated with depression.

Between March 2017 and February 2018, 31 healthy volunteers, 31 UC patients without depression, and 31 UC patients with depression from Longhua Hospital were enrolled. Clinical data and fecal samples were collected for each patient. Fecal bacteria were identified using 16 s rRNA sequencing. We compared microbial composition among the 3 groups using bioinformatic analysis.

Patients with UC with depression had higher disease severity (*P* < .05). The UC without depression group had moderate reduction of microbial abundance and uniformity compared to the control group. The UC with depression group had the lowest microbial abundance. With regard to the vital bacteria in the microbiota-gut-brain axis, patients with UC and depression had the lowest abundance of *Firmicutes*, *Clostridia*, and *Clostridiales* but the highest abundance of *Proteobacteria*, *Gammaproteobacteria*, and *Bacilli*.

The presence of depression in UC patients presented significant differences in the composition of gut microbiota compared with UC patients without depression, with increased abundance of *Firmicutes* and reduced abundance of *Proteobacteria.*

## 1 Introduction

Ulcerative colitis (UC) is an inflammatory bowel disease with unclear pathogenesis that causes inflammation and formation of ulcers in the colon and rectum. The incidence of UC has been consistently increasing in Asia recently.^[1,2]^ It was estimated that prevalence of UC in China has increased to 11.6 per 100,000 every year.^[3]^ Due to the disease's long duration and wide range of pathological changes, recurrences can sometimes occur even during remission, seriously affecting quality of life.^[4,5]^

The biological-psychological-social medical model has attracted more attention to the role of mental and psychological factors in the pathogenesis of UC. There is evidence that there is an interaction between ulcerative colitis activity and psychological disorders.^[6]^ It is widely accepted that patients with UC should be monitored for psychological well-being.^[7–11]^ The depression rate of UC patients ranges from 29% to 35%.^[12]^ In patients with recurrent UC, 60% of them display depression symptoms.^[13–16]^ Depression is a risk factor for UC recurrence and has a negative impact on quality of life.^[17]^

On the other hand, UC patients commonly experience changes to the composition of their gut microbiota. Some studies have found that UC is closely associated with gut microbiota disorders.^[18,19]^ Gut microbiota plays a significant role in the gut mucosal immune system.^[20]^ Scaldaferri et al found that the most severe inflammatory sites in the gut of UC patients were also the site with the highest abundance of bacteria.^[21,22]^ When the dominant bacterial species in the gut is altered, this results in instability of the gut microbiota and an immune reaction within the gut mucosa.^[23–25]^ Ghaisas et al revealed that microbial disorder can cause changes in the metabolism of bacteria, inducing gut inflammation.^[26]^ Changes to the innate characteristics of the gut microbiota can be used as a diagnostic marker and a prognosticator of UC.^[27]^

Emerging evidence has shown not only that the gut microbiota is important for intestinal physiology, but also that the microbiota-gut-brain axis (MGBA) strongly influences the central nervous system function and behavior.^[28–30]^ MGBA, a new concept, which encompasses the interactions between the microbiota, enteric nervous system, automatic nervous system, neuroimmune system, and central nervous system, was recently put forward.^[31–33]^ Interaction between the gut microbiota and peptide hormones may affect anxiety and depression.^[30]^ Normally, the intestinal barrier prevents translocation of bacterial products linking to the immune and nerve system. However, this barrier is suboptimal in inflammatory bowel disease patients, thereby allowing lipopolysaccharide to spread systemically and enhance peripheral and central inflammation and oxidative and nitrosative stress processes, including microglial, and astrocyte activation, with chronic glial activation contributing to a cascade of oxidative and nitrosative stress that can compromise neural function, thus causing depression.^[34,35]^ The metabolic substances produced by gut bacteria, such as phenylalanine, can have an impact on the nervous system.^[36]^

Despite the establishment of the links between ulcerative colitis (UC) and depression, between UC and gut microbiota, few correlations between depression and gut microbiota have yet been demonstrated especially in ulcerative colitis patients. According to a recent systematic review, all 6 included studies demonstrated differences in gut microbiota comparing major depressive disorder with control group. However, minimal consensus emerged regarding which bacterial taxa are most relevant to depression.^[37]^ The objective of our study was therefore to determine whether the comorbidity of depressive disorder in ulcerative colitis patients correlate with alterations in the gut microbiota and to identify the specific microbiota signatures associated with depression. We expect the findings can provide scientific evidence for precise clinical diagnosis and the treatment of gut microbiota abnormalities in UC patients with depression.

## 2 Patients and methods

### 2.1 Patient recruitment

All participants were required to sign an informed consent form prior to enrollment. UC patients in Longhua Hospital were enrolled from March 2017 to February 2018. Endoscopy and histological examination and clinical symptoms of patients provided an objective basis for the diagnosis of UC. The clinical features examined included: the recurring diarrhoea (which may contain blood, mucus or pus), abdominal pain (needing to empty your bowels frequently), tenesmus, severe fever and extra intestinal manifestation. The endoscopic features examined included: loss of vascular pattern, erythema, granularity, friability, erosions, ulcerations, and spontaneous bleeding.^[38,39]^ The severity of UC activity was graded based on the Mayo criteria.^[40]^ The inclusion criteria required patients to be:

(1) UC patients,
(2) aged 18 to 70,
(3) able to communicate normally and finish questionnaires,
(4) volunteers who agreed to participate in the study and signed informed consent.

The exclusion criteria included:

(1) severe complications, such as megacolon, enteroparalysis, intestinal perforation, or obstruction,
(2) history of malignant tumor,
(3) autoimmune disease,
(4) severe complications in the heart, brain, or blood,
(5) bacterial dysentery, colitis, or other diseases in the gastrointestinal system,
(6) microecologic or antibiotic treatment in the previous 2 weeks,
(7) pregnancy or lactation,
(8) refusal to give informed consent.

Healthy volunteers were also included as a control group. The healthy subjects were recruited in this hospital from the physical examination records, the items of which include colonoscopy as well as inquiries and collection of personal medical history. A self-rating depression scale (SDS) was used to evaluate levels of depression.^[35]^ The raw scores of the 20 questions in the scale were converting to index scores, and an index score of 53 and over is classified as depressive disorder in Chinese population.^[41,42]^ Two senior psychiatrists evaluated the healthy control group to verify there were no signs of depression. Clinical data was collected from medical records. This study protocol was approved by the Ethics Committee of Longhua Hospital affiliated to Shanghai University of Traditional Chinese Medicine (March 4th 2015).

### 2.2 Fecal sample collection and DNA extraction

A sterile plastic cup was used to collect the samples for bacterial genomic DNA extraction, stored in an icebox, then delivered to the laboratory at −80°C. A total of 200 mg feces were collected to extract the fecal microbial DNA via the DNA Stool Extraction Kit (SIGMA, Germany). A NanoDrop ND-1000 spectrophotometer (Thermo Electron) was used to quantify DNA. 1.0% agarose gel electrophoresis on gels containing 0.5 mg/mL ethidium bromide was used to assess the integrity and size of DNA. DNA was stored at −20°C before analysis.

### 2.3 16 s rRNA sequencing and PCR amplification

Each sample was tested by triplicate PCR reactions. The bacterial genomic DNA was amplified with the 338F: 5’-ACTCCTACGGGAGGCAGCAG-3’and 806R: 5’-GGACTACHVGGGTWTCTAAT-3 primers specific for the V3-V4 hyper variable regions of the 16S rRNA gene. The PCR reaction was set up using 2 μL DNA extraction sample, 0.4 μL of each primer (338F and 806R, 10 pmol/μL), and 0.2 μL Taq DNA polymerase (5 units/μL). The thermocycling conditions were denaturation at 95°C for 5 min initially; and for 35 total cycles, denaturation was at 95°C for 30 seconds, annealing was at 60°C for 30 seconds, elongation was at 72°C for 1 minutes; and final extension was at 72°C for 10 minutes. Gel electrophoresis was used to evaluate the results as described previously.^[43]^

### 2.4 Bioinformatics and statistical analysis

Chi-square statistical tests were used to compare categorical variables of the clinical features among UC without depression, UC with depression, and the control groups. T-tests or ANOVA were used to compare the continuous variables. Titanium PyroNoise software was used to denoise the raw pyrosequencing reads obtained from the sequencer. Using a combination of tools from Mothur (ver. 1.25.0; http://www.mothur.org) and custom Perl scripts, the resulting pyrosequencing reads were filtered according to the barcode and primer sequences. Chimera sequences arising from the PCR amplification were detected via Chimera Slayer and excluded from the denoised sequences. Each sample was assigned to the high-quality sequences according to the barcode. Mothur was used to cluster the high-quality reads into operational taxonomic units (OTUs). When the OTUs reached a 97% nucleotide similarity level, they were used for alpha diversity (Shannon, Simpson, and evenness indices), richness (ACE and Chao1), Good's coverage, Venn diagram, and rarefaction curve analyses via Mothur. Using the RDP Bayesian Classifier, Taxonomy-based analyses were performed by classifying each sequence. Spearman's rank-correlation analysis was used to calculate the correlations between variables. SPSS (ver. 23.0) data analysis software (SPSS Inc., Chicago, IL) was used to perform the statistical analyses. A *P* value < .05 was considered to indicate statistical significance.

## 3 Results

### 3.1 Clinical features

There were 31 patients in each of the 3 groups: UC with depression, UC without depression, and healthy control. Clinical data were shown in Table 1. There were no statistically significant differences between the groups in terms of demographic data, disease duration, clinical type and extent. However, UC patients with depression had worse UC disease severity than UC patients without depression. Fourteen patients (45%) in the UC with depression group had severe UC disease symptoms, while only 4 patients (13%) in the UC without depression group had severe UC disease symptoms.

**Table 1:** Demographic and clinical data of patients with ulcerative colitis.

	Control group	UC without depression	UC with depression	*P*
Sex				.958
Male	16 (52)	17 (55)	16 (52)	
Female	15 (48)	14 (45)	15 (48)	
Age	43.13 ± 10.98	44.16 ± 11.49	41.94 ± 13.42	.923
BMI	22.4 ± 2.6	22.8 ± 1.9	22.1 ± 2.1	.406
Disease duration				.888
< 1 yr		5 (16)	6 (19)	
1–3 yr		10 (32)	11 (35)	
3–5 yr		5 (16)	3 (10)	
> 5 yr		11 (35)	11 (35)	
Clinical type				1.00
incipient		2 (6)	2 (6)	
chronic		29 (94)	29 (94)	
Extent				.824
Ulcerative proctitis		2 (6)	3 (10)	
Left colon		15 (48)	16 (52)	
extensive		14 (45)	12 (39)	
Severity				.006
mild		23 (74)	11 (35)	
moderate		4 (13)	6 (19)	
severe		4 (13)	14 (45)	

### 3.2 Gut microbiota analysis

**Figure 1:**
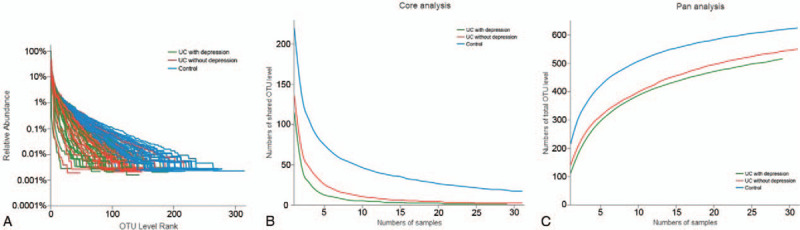
Comparison of the abundance of bacteria among the 3 groups. A: Rank-Abundance curve; B: Core-species analysis; C: Pan-species analysis.

Out of the 93 fecal samples amplified by PCR, 91 of the samples were qualified for further analysis. Two samples in the UC with depression group were excluded because of unqualified fecal sample. Supplemental Table 1 shows the microbial community composition of all the samples. Only 1 domain and 1 kingdom were present. The number of phyla, classes, orders, families, genera, and species were 9, 17, 24, 45, 159, and 311, respectively. Figure 1 shows the abundance and uniformity of species in each of the 3 groups. The control group had the highest abundance and uniformity. The UC with depression group had the lowest abundance.

**Figure 2:**
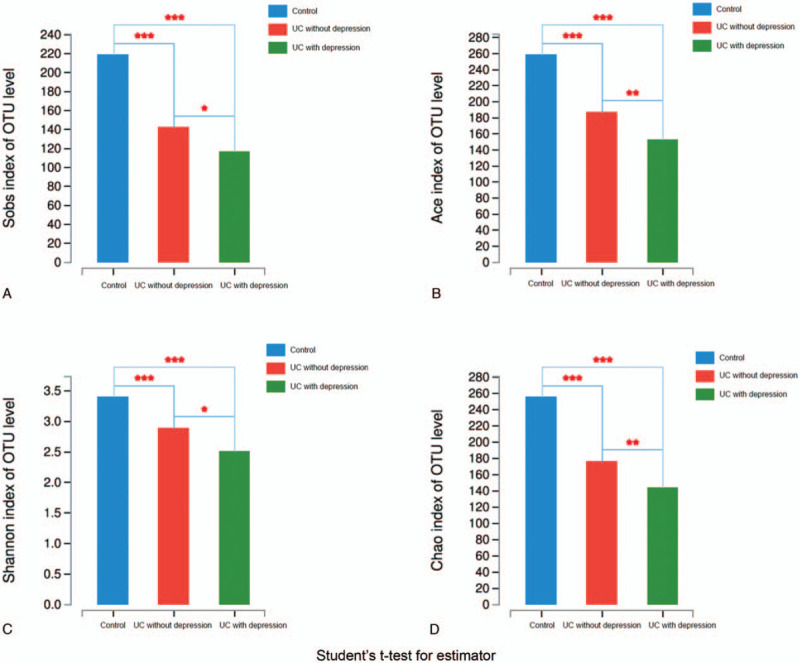
Pairwise comparison among groups using Sob, Ace, Shannon and Chao indices. A: Sobs index of OUT lever; B: Ace index of OUT lever; C: Shannon index of OUT lever; D: Chao index of OUT lever. ^∗^*P* ≤.05, ^∗∗^*P* ≤ .01, ^∗∗∗^*P* ≤ .001.

The UC with depression group also had the lowest pan-species and core-species values. Species in the UC without depression group were slightly higher than in the UC with depression group, but much lower than in the control group. Ace, Chao, Sob, and Shannon indices were included in our alpha diversity analysis. Ace, Chao, and Sob indices indicated the abundance of the samples (Supplemental Figure 1). The pair wise comparison of Ace, Chao, Sob, and Shannon indices among the control group, UC without depression group, and UC with depression group had statistical significance (Fig. 2). The control group had the highest abundance and the UC with depression group had the lowest abundance. The same order was observed using the Shannon index for diversity (Supplemental Figure 2).

**Figure 3:**
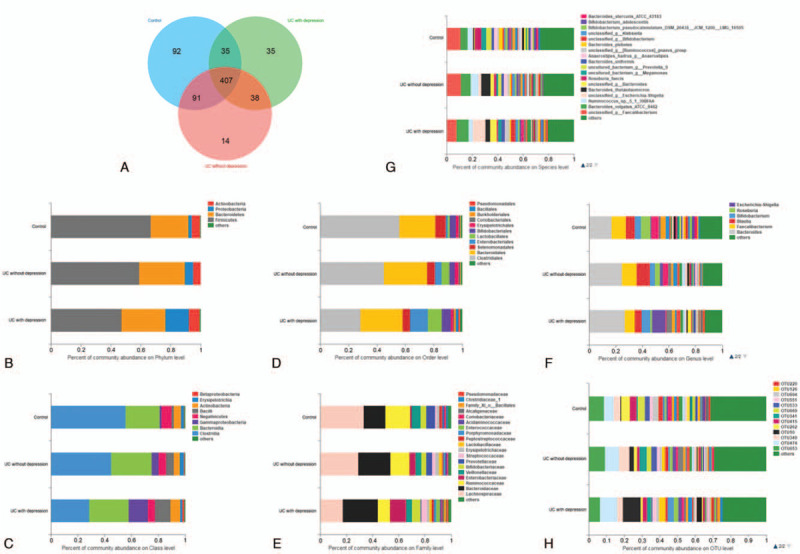
Composition of the gut microbiota at different levels. A: Venn diagram at OTU level; B: Histogram at the phylum level; C: Histogram at the class level; D: Histogram at the order level; E: Histogram at the family level; F: Histogram at the genus level; G: Histogram at the family level; H: Histogram at the OTU level.

**Figure 4:**
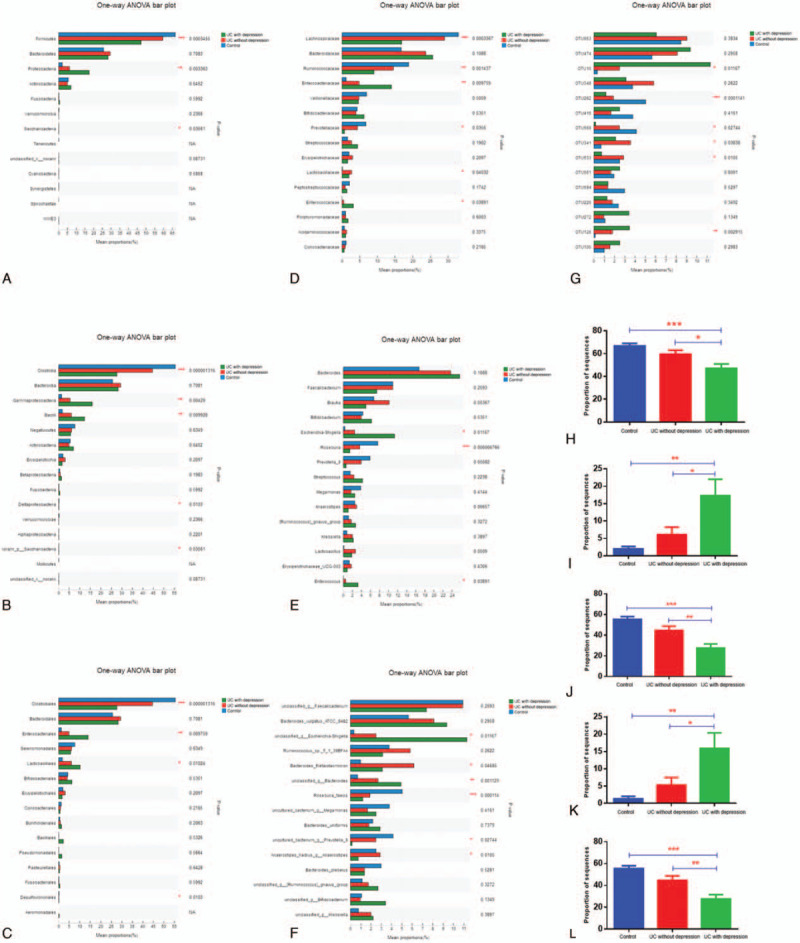
Comparison of the gut microbiota at different levels. A: Phylum level; B: Class level; C: Order level; D: Family level; E: Genus level; F: Family level; G:OTU level; H: Firmicutes at the phylum level; I: Proteobacteria at the phylum level; J: Clostridia at the class level; K: Gammaproteobacteria at the class level; I: Clostridiales at the order level. ^∗^*P* ≤ .05, ^∗∗^*P* ≤.01, ^∗∗∗^*P*≤ .001.

Figure 3 shows the microbiota composition of each of the 3 groups. There were 407 OTUs shared by all 3 groups. The control group had 92 unique OTUs, the UC without depression group had 14 unique OTUs, and the UC with depression group had 35 unique OTUs. Figure 4 shows the results of the pairwise comparison. At the phylum level, the control group had the highest abundance of *Firmicutes*. The UC without depression group had the second highest abundance and the UC with depression group had the lowest abundance (*P* < .001). In contrast, the control group had the lowest abundance of *Proteobacteria*. The UC with depression group had the highest abundance of *Proteobacteria* (*P* < .01). At the class level, the control group had the highest abundance of *Clostridia*. The UC with depression group had the lowest abundance (*P* < .001). In contrast, the control group had the lowest abundance of *Gammaproteobacteria* and *Bacilli*. The UC with depression group had the highest abundance of *Gammaproteobacteria* and *Bacilli* (*P* < .01). At the order level, the control group had the highest abundance of *Clostridiales*. The UC with depression group had the lowest abundance (*P* < .001). In contrast, the control group had the lowest abundance of *Enterobacteriales* and *Lactobacillales*. The UC with depression group had the highest abundance of *Enterobacteriales* (*P* < .01) and *Lactobacillales* (*P* < .05). At the family level, the control group had the highest abundance of *Lachnospiraceae* (*P* < .001) and *Prevotellaceae* (*P* < .05). The UC with depression group had the lowest abundance of each of these families. In contrast, the control group had the lowest abundance of *Ruminococcaceae* and *Enterobacteriaceae*. The UC with depression group had the highest abundance of *Ruminococcaceae* (*P* < .01) and *Enterobacteriaceae* (*P* < .01). At the genus level, the control group had the highest abundance of *Roseburia* (*P* < .001). The UC with depression group had the lowest abundance of *Roseburia*. In contrast, the control group had the lowest abundance of *Escherichia-Shigella* and *Enterococcus*. The UC with depression group had the highest abundance of *Escherichia-Shigella* (*P* < .05) and *Enterococcus* (*P* < .05). At the species level, several bacteria were present among the 3 groups at statistically different amounts. These included an unclassified *Escherichia-Shigella* species, *Bacteroides thetaiotaomicron*, an unclassified *Bacteroides* species, *Anaerostipes hadrus*, *Roseburia_faecis*, and an uncultured *Prevotella* 9 species.

## 4 Discussion

This study found significant differences in disease severity comparing UC patients with and without depression. With regard to gut microbiota, UC with depression was observed with lowest abundance while healthy controls had the highest abundance. Furthermore, there were statistically differences in the levels of specific bacterial taxa in gut microbiota associated with depression in UC patients.

In our study, UC with depression was found associated with more severe disease symptoms. Our findings were consistent with previous reports that depression is associated with the disease severity. A prospective cohort study concluded that long-term perceived stress triggers subsequent exacerbation in ulcerative colitis.^[44]^ In patients with inflammatory bowel disease, disease activity had a significant association with increased risk of depression.^[45]^ However, according to an analysis based on the data from the Nurse's Health Study, baseline and subsequent depressive symptoms increased the risk for Crohn's disease but not for ulcerative colitis. The contradictory results possibly arose from measures at different time points.

Our study investigated the microbiota in fecal samples of UC patients and healthy population. We found that microbial abundance and diversity were different among the healthy control group, the UC without depression group, and the UC with depression group. The abundance and diversity of the gut microbiota in UC patients were lower than that in healthy persons. Compared to healthy controls, ulcerative colitis presented increased *Firmicutes* and decreased *Bacteroidetes*. Furthermore, the UC with depression group displayed more severe disorder of the microbiota. In the UC with depression group, the abundance of *Firmicutes*, *Clostridia*, and *Clostridiales* was lower, and the abundance of *Proteobacteria* and *Gammaproteobacteria* was higher.

Until now, there has been controversy surrounding the relationship between gut microbiota diversity and the central nervous system. Naseribafrouei et al found that there is no difference in gut microbiota diversity between depression patients and healthy patients.^[46]^ However, our results showed that the presence of depression decreased microbiota diversity in UC patients. The alteration of microbiota diversity in UC without depression patients was different from that in UC with depression patients. At the phylum level, the abundance of *Firmicutes* was lower in the UC with depression group. *Firmicutes* synthesize short-chain fatty acids, such as acetate and butyrate, and provide nutritional substances to gut epithelial cells.^[47]^ Short-chain fatty acids induce immune tolerance of mucosa and B cell differentiation.^[48]^ Butyrate provides energy to the gut mucosa and reduces the inflammatory response.^[49]^ When the number of butyrate-producing bacteria decreases, the incidence of gut inflammation increases.^[49]^ Adherent-invasive is a type of *Proteobacteria*. adherent-invasive *Escherichia coli* adheres to gut epithelial cells using microtubular polymerization, inducing secretion of an inflammation factor.^[50]^ They also replicate in the J774-A1 macrophage cell line and promote macrophages to secrete TNF.^[51]^ The changes in abundance of *Firmicutes* and *Proteobacteria* may have a correlation with UC and depression. The levels of *Firmicutes* and *Proteobacteria* were reportedly being associated with relieving psychiatric disorders by alterations in amino acid metabolism and evaluation of bile acid biosynthesis.^[52,53]^ Zhu HZ et al found that regulating the abundance of *Proteobacteria*, *Firmicutes* and *Bacteroidetes* can improve depression-like behavior which involved in this process may be related to short-chain fatty acids, lipopolysaccharides, and intestinal inflammation.^[54]^ Zhang XJ et al found that changing of the abundance of *Bacteroidetes*, *Proteobacteria* and *Firmicutes* which are involved in tryptophan catabolism might aggravate intestinal injury in DSS-induced colitis in mice.^[55]^ Some scholars have found that altering the diversity and composition of gut microbiota which modulate T cell repertories and regulate T-helper cell balance(Th1/Th2 balance, Th17, Treg) might contribute to the therapeutic strategies in UC.^[56,57]^ Findings suggest that gut microbiota and inflammation may be differentially associated with mood disorder via the MGBA model.^[58]^ UC can be accompanied by depression and microbial disorder. According to the MGBA model, depression can change the composition of bacteria in UC patients. Modifying the gut microbiota may improve depression symptoms and mental intervention may be beneficial to UC patients.^[59,60]^

However, our study also had some disadvantages. The sample size was relatively small. Besides, although we have made efforts to control potential confounders, there may still exist many factors that could affect our results, for instance, the administration of antipsychotics and diet might contribute to the alterations in the gut microbiota. To improve the reliability of results, similar studies with larger sample sizes and stricter inclusion criteria are essential. In addition, the temporal and causal relationship between gut microbiota and depression has not been elucidated in our study. Prospective studies examining both the baseline and subsequent composition of gut microbiota in UC patients with and without depression are warranted in the future.

In conclusion, our study compared the clinical features and microbial composition of fecal samples of UC patients with and without depression, as well as healthy controls by 16s-rRNA sequencing. The presence of depression in UC patients presented significant differences in the composition of gut microbiota compared with UC patients without depression, with increased abundance of *Firmicutes* and reduced abundance of *Proteobacteria.*

## Author contributions

**Conceptualization:** De-Liang Chen, Yan-Cheng Dai, Ya-Li Zhang, Zhipeng Tang.

**Data curation:** De-Liang Chen, Yan-Cheng Dai, Lie Zheng, You-Lan Chen, Ya-Li Zhang.

**Formal analysis:** Lie Zheng, You-Lan Chen, Ya-Li Zhang.

**Supervision:** Zhipeng Tang.

**Writing – original draft:** De-Liang Chen, Yan-Cheng Dai.

**Writing – review & editing:** De-Liang Chen, Yan-Cheng Dai, Ya-Li Zhang, Zhipeng Tang.

## Supplementary Material

Supplemental Digital Content

## Supplementary Material

Supplemental Digital Content

## Supplementary Material

Supplemental Digital Content
