# Supplementary material for: Features of the gut microbiota in ulcerative colitis patients with depression: A pilot study
Source: Medicine (Baltimore). 2021 Feb 19;100(7):e24845. doi: 10.1097/MD.0000000000024845 (PMC7899815; doi:10.1097/MD.0000000000024845)
Supplement: Supplemental Digital Content [file medi-100-e24845-s001.docx]

Table S1. The microbial community composition of all the samples.

|  | domain | kingdom | phylum | class | order | family | genus | species | OTU |
| --- | --- | --- | --- | --- | --- | --- | --- | --- | --- |
| No. | 1 | 1 | 9 | 17 | 24 | 45 | 159 | 311 | 419 |
