# Supplementary figures and images for: Features of the gut microbiota in ulcerative colitis patients with depression: A pilot study
Source: Medicine (Baltimore). 2021 Feb 19;100(7):e24845. doi: 10.1097/MD.0000000000024845 (PMC7899815; doi:10.1097/MD.0000000000024845)

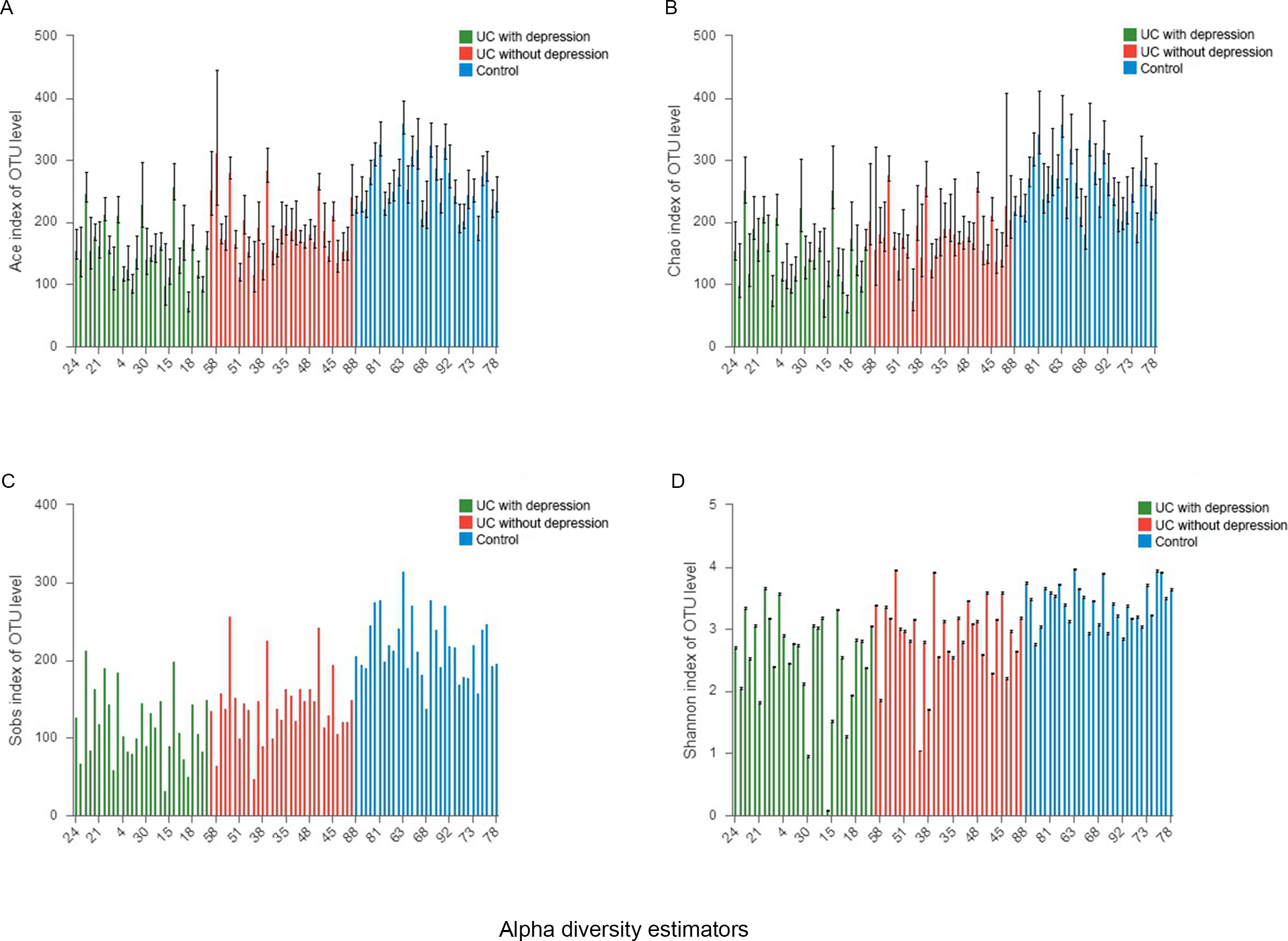

Supplement: Supplemental Digital Content [file medi-100-e24845-s002.tif]

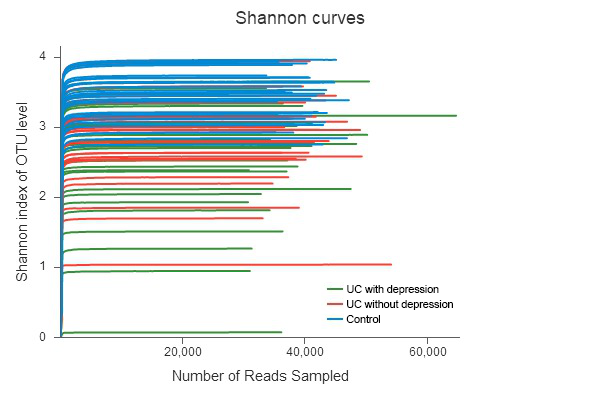

Supplement: Supplemental Digital Content [file medi-100-e24845-s003.tif]
